# Polycrystalline WO_3−x_ Thin Films Obtained by Reactive DC Sputtering at Room Temperature

**DOI:** 10.3390/ma16041359

**Published:** 2023-02-06

**Authors:** Cecilia Guillén

**Affiliations:** Centro de Investigaciones Energéticas Medioambientales y Tecnológicas (CIEMAT), Avda. Complutense 40, 28040 Madrid, Spain; c.guillen@ciemat.es

**Keywords:** metal oxide, crystallinity, optical absorption, electrical conductivity

## Abstract

Tungsten oxide thin films have applications in various energy-related devices owing to their versatile semiconductor properties, which depend on the oxygen content and crystalline state. The concentration of electrons increases with intrinsic defects such as oxygen vacancies, which create new absorption bands that give rise to colored films. Disorders in the crystal structure produce additional changes in the electrical and optical characteristics. Here, WO_3−x_ thin films are prepared on unheated glass substrates by reactive DC sputtering from a pure metal target, using the discharge power and the oxygen-to-argon pressure ratio as control parameters. A transition from amorphous to polycrystalline state is obtained by increasing the sputtering power and adjusting the oxygen content. The surface roughness is higher and the bandgap energy is lower for polycrystalline layers than for amorphous ones. Moreover, the electrical conductivity and sub-bandgap absorption increase as the oxygen content decreases.

## 1. Introduction

Tungsten oxides are naturally abundant, environmentally friendly, and their semiconductor properties can be tuned according to oxygen content and crystalline state [1]. Furthermore, they are easily produced in the form of thin films or coatings, making them special candidates for a variety of energy-related applications, such as smart windows [1], batteries [2], photocatalysis [3] or solar cells [4], and also for faster gas sensors [5].

WO_3_ bulk crystals are built up from [WO_6_]^6−^ octahedra, where the tungsten atom is placed in the center of the octahedra with the oxidation state W^6+^. The ideal WO_3_ crystal consists of ordered networks of corner-sharing octahedra, but in practice, different phases can be obtained due to atomic displacements and rotations, which reduce the crystal symmetry. Monoclinic WO_3_ is the most stable phase at room temperature (17 °C < T < 330 °C), converting to orthorhombic and tetragonal symmetries at 330 °C and 740 °C, respectively [1]. These crystalline phases can be described as distortions of the cubic ReO_3_-type structure, explained by the low formation energy for defects in the cubic lattice [6]. If WO_3_ is reduced, oxygen loss induces new structural features, giving rise to non-stoichiometric phases WO_3−x_ (0 < x < 0.4) where blocks of corner-sharing octahedra are connected by edge sharing [2].

Perfect WO_3_ is a transparent insulating semiconductor with a bandgap energy of 2.7 eV [3], but in reality the electrical and optical characteristics of tungsten oxides are highly dependent on their crystalline structure and oxygen content. The electron concentration can be increased by intrinsic defects such as oxygen vacancies (V_O_^2+^), which create new absorption bands below the conduction band and, as a result, color appears from bluish to dark gray [7]. For technological applications, thin films of WO_3−x_ are particularly attractive, considering that additional changes in electrical and optical characteristics can be achieved by reducing the crystal size into the nanometer range [8].

Sputtering has been widely used to prepare tungsten oxide thin films with controllable microstructures and tunable physical properties. Moreover, reactive DC sputtering via a metallic W target provides the advantages of low cost and high deposition rates [9]. It is known that amorphous tungsten oxide coatings are commonly achieved by DC sputtering on unheated substrates [10,11,12]. Subsequent heating in the 400–500 °C range is needed to achieve crystallization in the monoclinic WO_3_ [10] or monoclinic W_18_O_49_ [11] structures. Otherwise, a stable orthorhombic WO_3_ phase has also been obtained from amorphous layers grown under a low oxygen content atmosphere, by subsequent annealing in air at 400–600 °C [12]. Only small WO_3_ crystallinity has been reported for low substrate temperatures (≤200 °C), highly dependent on the sputtering power [13], the working pressure [14] and the oxygen partial pressure [15].

In this work, polycrystalline WO_3−x_ thin films consisting of WO_3_ together with W_3_O_8_ or W_18_O_49_ crystalline phases are obtained directly on unheated glass substrates by reactive DC sputtering from a pure metal target. A transition from the amorphous state to orthorhombic or monoclinic symmetries is achieved by increasing the sputtering power and the oxygen-to-argon pressure ratio. The structure, morphology and optical and electrical characteristics of the samples are analyzed as a function of the preparation conditions to determine the different effects of crystallinity and oxygen vacancy defects.

## 2. Materials and Methods

Tungsten oxide layers were prepared on unheated soda-lime glass substrates by reactive DC magnetron sputtering from a W target (purity 99.99%) of 15 cm in diameter. The chamber was evacuated to a base pressure of 4 × 10^−4^ Pa before introducing a fixed argon flow of 60 sccm and an adjusted oxygen flow in the 3–30 sccm range, using independent mass flow controllers. The oxygen flow was varied to achieve different oxygen partial pressures, O_pp_ (%) = 100 p(O_2_)/[p(O_2_) + p(Ar)], in the range 5–30%. Then, a DC source was connected to the target, operating at a constant power value set to 200 or 400 W, resulting in a power density of 1 W/cm^2^ or 2 W/cm^2^, respectively. The growth rate was about 25 nm/min at the lowest power density and about 50 nm/min at the highest, with only minor variations at different oxygen pressures in the present range. For each case, the deposition time was adjusted to obtain a same film thickness of 300 ± 20 nm.

**Figure 1:**
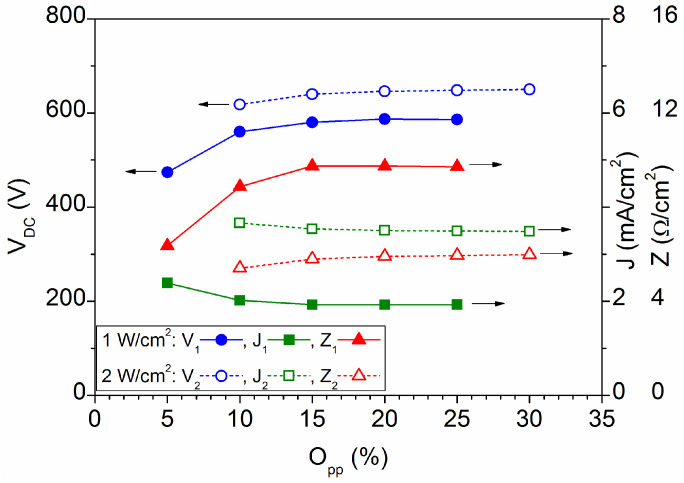
Discharge characteristics as a function of oxygen percentage for the two power values applied to the sputtering target.

Figure 1 shows the current, voltage and impedance characteristics as a function of oxygen percentage for the two power values applied to the target. The range studied goes from 5% to 30% O_pp_, which corresponds to oxygen gas inputs from 4 sccm to 30 sccm. Therefore, all processes were carried out in the oxide operating mode of the target. The impedance of the discharge increases with the increase of the oxygen content in the chamber, which is attributed to a greater capture of electrons from the plasma due to the high electronegativity of oxygen [16]. Otherwise, the impedance is always lower when the applied power is higher, which is related to a better sputtering efficiency due to an important increase of the ion current in the plasma at high power [17]. The discharge current is the sum of the ion current (I_i_) and the electron current (I_e_), being I_i_ increasing with the sputtering power, while I_e_ decreases when the oxygen percentage increases.

The crystallographic properties of the samples have been examined by X-ray diffraction (XRD) with radiation Cu K-α1 (λ = 1.54056 Å) in a Philips X’pert instrument, using Bragg–Brentano θ−2θ configuration. The standard powder diffraction files (PDF) were used to identify the crystalline phases in the diffractograms. Elemental compositions were obtained by electron probe micro-analysis in a JEOL JSM-6400 operating at 20 kV. The topography was examined by atomic force microscopy (AFM) with a Park XE-100, which allows surface roughness to be quantified from digital images. The optical characterization uses transmittance (T) and reflectance (R) measurements performed with a Perkin–Elmer Lambda 9 double beam spectrophotometer, in a wavelength range from 250 to 2500 nm and taking the air as reference. The transmittance is corrected for reflection losses, T_c_ (%) = 100 T(%)/(100 − R(%)), and the optical absorption coefficient is calculated as [18,19]: α = (1/t) ln[100/T_c_(%)], including the film thickness value (t) obtained with a Dektak 3030 profilometer. The electrical conductivity of the layers has been determined with a four-point probe system Veeco FPP5000.

## 3. Results and Discussion

The XRD patterns measured for the various samples have proven that the tungsten oxide films prepared at P = 2 W/cm^2^ and O_pp_ = 20–30% have a polycrystalline structure, while all other layers are amorphous, as indicated by absence of diffraction peaks in their respective patterns. The data summarized in Table 1 indicate that both the sputtering power and the oxygen pressure influence the composition and crystallinity of the tungsten oxide coatings, in a similar way to that observed in other works [13,15,20]. Films may grow amorphous due to high density of oxygen vacancies [7] or low energy of the particles impinging on the substrate [19].

**Figure 2:**
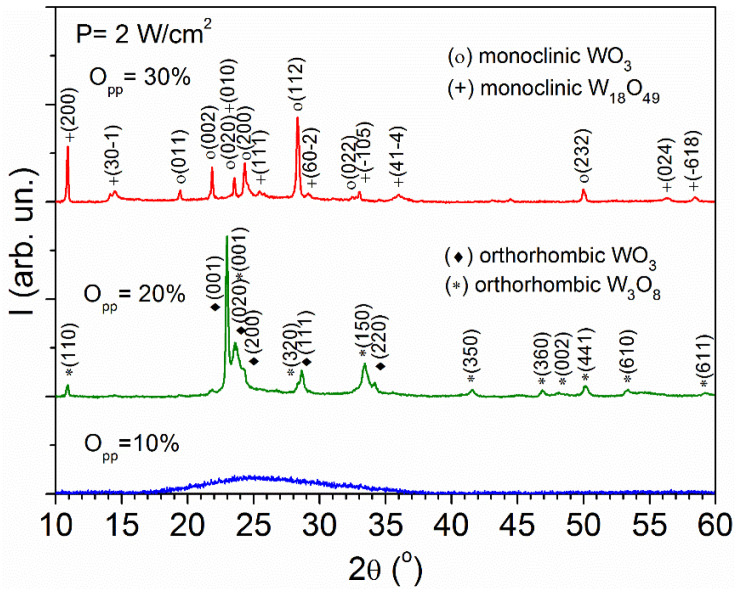
XRD patterns corresponding to WO_3−x_ thin films prepared by reactive sputtering at 2 W/cm^2^ and different oxygen partial pressures.

According to Figure 2, the layer grown at P = 2 W/cm^2^ and O_pp_ = 20% exhibits XRD peaks that correspond to orthorhombic WO_3_ (PDF#20-1324) and orthorhombic W_3_O_8_ (PDF#65-1175). The strongest peaks are recorded at 2θ = 22.94° and 2θ = 23.54°, assigned to the (001) planes of WO_3_ and W_3_O_8_, respectively. Otherwise, films prepared at P = 2 W/cm^2^ and O_pp_ = 30% show a mixture of monoclinic WO_3_ (PDF#83-0950) with a strong (112) peak at 2θ = 28.33°, and monoclinic W_18_O_49_ (PDF#36-0101) with strong (200) peak at 2θ = 10.91°. It is known that monoclinic is the most stable WO_3_ phase at room temperature and symmetries higher than the monoclinic may result from static disorder [6], attributed to the presence of clusters of edge-sharing octahedra randomly distributed in the lattice. Such a tendency is also found in the oxygen-deficient WO_3−x_ phases, but in this case the edge-sharing octahedra are not randomly distributed since they are in periodic patterns. Specifically, the W_3_O_8_ structure contains groups of four edge-sharing WO_6_ octahedra mutually linked by corner-sharing with single octahedra [21]. Otherwise, the W_18_O_49_ structure contains both octahedral and pentagonal bipyramidal coordination of the metal atoms by oxygen [22]. Analogous W_3_O_8_ and W_18_O_49_ phases have been obtained by reduction of tungsten trioxide or by heating appropriate mixtures of W and WO_3_ [21,22,23].

**Figure 3:**
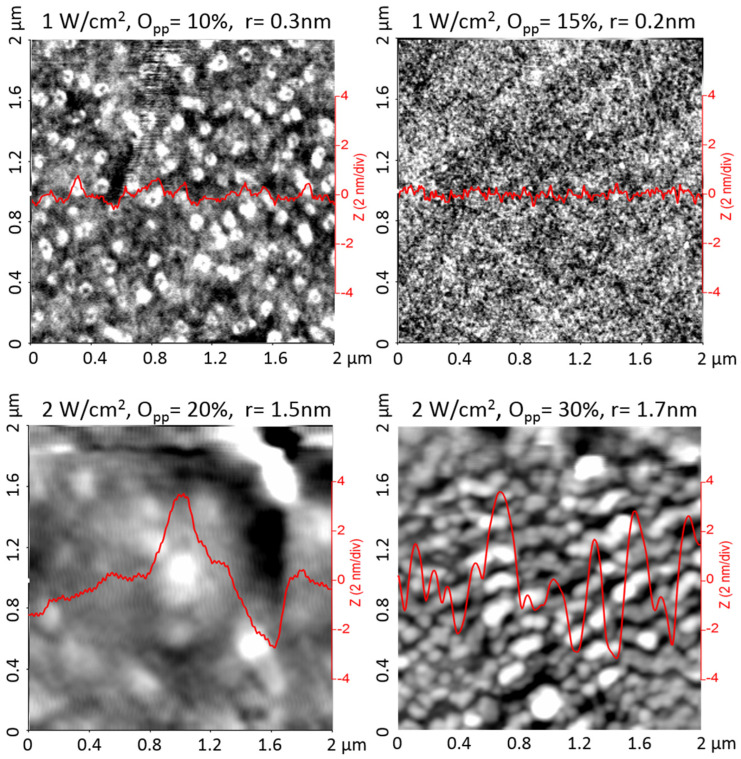
AFM images and representative line scans taken on the surface of WO_3−x_ layers prepared at different sputtering powers and oxygen partial pressures. The root-mean-square roughness value (r) is included for each sample.

The surface morphology and corresponding root-mean-square roughness value (r) of the samples are illustrated in Figure 3, by representative AFM images on 2 μm × 2 μm areas. A line scan taken in the middle of each image is included for comparison. It can be seen that the amorphous layers grown at P = 1 W/cm^2^ are constituted by small grains that give smooth surfaces, with r ≤ 0.3 nm. Increasing the sputtering power to P = 2 W/cm^2^ promotes the growth of larger grains that clump together to form cauliflower-like clusters, with a significant increment of the surface roughness to r ≥ 1.5 nm. This is related to the crystallization of the layers (Figure 2), since by increasing the sputtering power, the deposited particles can obtain enough energy to migrate along the surface and inside, which favors the formation of crystalline structures and larger clusters that increase the surface roughness. A similar increment in the root-mean-square roughness from amorphous to polycrystalline films has been reported for other sputtered WO_3_ coatings [10,12].

**Figure 4:**
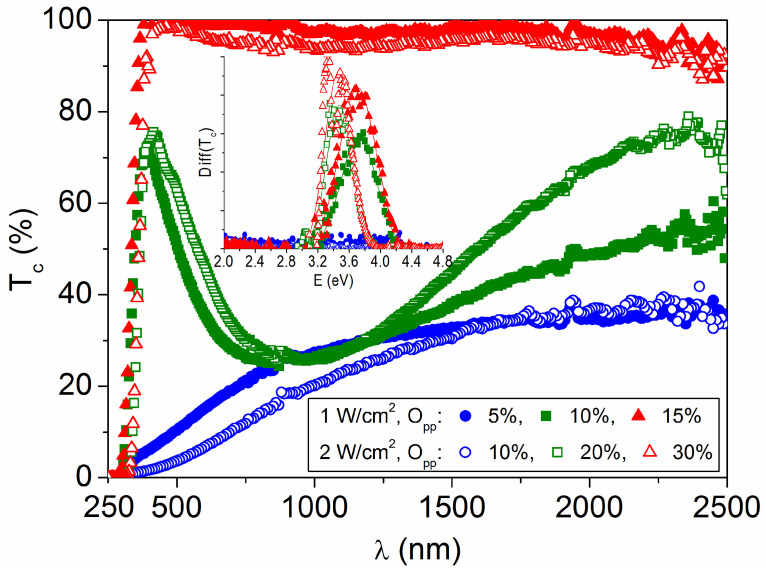
Optical transmittance as a function of the light wavelength for WO_3−x_ films prepared at different sputtering powers and oxygen partial pressures. The inset shows differential of the transmittance curves as a function of the light energy (from the derivative dT_c_/dE).

The optical transmittance of the tungsten oxide films is plotted in Figure 4 as a function of the light wavelength. For each sputtering power, the layers prepared at high oxygen pressures are transparent in the visible and near-infrared regions, as expected for stoichiometric WO_3_ [19], but the transmittance decreases when the oxygen content decreases. The strong transmittance decline in the near infrared (green plots in Figure 4) and also in the visible range (blue plots in Figure 4) indicates the presence of oxygen vacancy defects (V_O_^+^ and/or V_O_^2+^) together with tungsten atoms in a lower oxidation state (W^5+^ and/or W^4+^), which are responsible for sub-bandgap absorption [18,24]. Such intrinsic defects can be tuned by the preparation conditions, taking into account that a higher discharge power can sputter more metal atoms requiring higher oxygen content in the chamber to achieve near-stoichiometric ratios, as it is evidenced by the transmittance spectra and the other data in Table 1.

The bandgap energy (E_g_) for each sample is estimated as the inflection point of the respective transmittance curve [25], the peak of the derivative dT_c_/dE, which is included in the Figure 4 inset. This is a good approximation for the bandgap of extended states, when the edges of the valence and conduction bands are not abrupt, and the tail states complicate the optical transitions [26]. The layers with the lowest oxygen content (O/W ~ 2.60 atomic ratio) have a metallic behavior that prevents bandgap determination. A semiconductor-to-metal transition is expected in WO_3−x_ by increasing the oxygen deficiency, when the Fermi energy falls in a continuum of electronic states due to the increment of intrinsic defects [27]. The coloration (visible absorption) is accompanied by a proportional change in the electrical conductivity of the samples, which is given in Table 1, because the oxygen vacancy defects are acting as electron donors [24]. The metallic films are amorphous regardless of the sputtering power used. Other amorphous layers, prepared at P = 1 W/cm^2^ and O_pp_ ≥ 10%, show E_g_ = 3.71 ± 0.1 eV, while polycrystalline coatings prepared at P = 2 W/cm^2^ and O_pp_ ≥ 20% show a lower E_g_ = 3.46 ± 0.1 eV. These values are in accordance to those reported by other authors, being generally higher for amorphous WO_3−x_ layers than for polycrystalline ones [10,28], and clearly superior to the bandgap of bulk WO_3_, which is 2.71 eV [7]. In disordered films, bandgap widening has been attributed to quantum confinement, since the length over which the electrons can move freely is limited [29].

**Figure 5:**
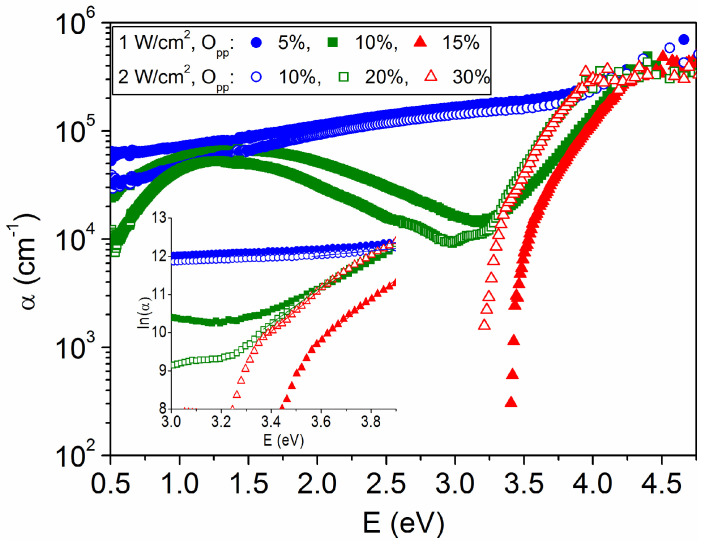
Optical absorption coefficient as a function of light energy for the WO_3−x_ samples depicted in Figure 4. In the inset, the Urbach energy (E_U_) is calculated from the inverse of the slope of ln(α) vs. E.

The influence of defects and disorder on the optical characteristics is also evaluated from the absorption spectra plotted in Figure 5 for the various WO_3−x_ films. At light energies just below the bandgap value (E < E_g_), the extent of the absorption edge is characterized by the Urbach energy, E_U_, which indicates the width of the band tails of the localized states within the bandgap region. The calculations in the Figure 5 inset prove that the experimental data follow Urbach’s rule [30]: α = α_0_ exp(E/E_U_), with the respective E_U_ values listed in Table 1. The highest Urbach energies (E_U_ > 1.5 eV) correspond to the amorphous films with metallic character, which show a residual absorption (α > 0) throughout the measured spectrum and are brown in color. Otherwise, the Urbach energy is minimal (E_U_ = 0.18 ± 0.01 eV) for the transparent layers close to WO_3_ stoichiometry, with no significant difference between their amorphous or polycrystalline nature, as observed for other WO_3_ films [31,32]. In the intermediate conditions, the samples exhibit slightly higher values (E_U_ = 0.21–0.29 eV) and bluish-gray color due to the selective absorption band located around 1.4 eV, which is typical of WO_3−x_ coatings with x = 0.2–0.3 [20]. Therefore, E_U_ values depend mainly on the oxygen vacancy states, as has been found in previous works [30,31].

## 4. Conclusions

The composition, structure and physical properties of reactively sputtered WO_3−x_ films can be tuned by discharge power and oxygen partial pressure. The films grow amorphous when the energy of the particles impinging on the substrate is not high enough or when there are high oxygen vacancy densities due to low oxygen content (O/W ≤ 2.6). Increasing the sputtering power and the oxygen pressure makes it possible to obtain a mixture of orthorhombic WO_3_ and W_3_O_8_ with an overall composition O/W = 2.82 or a mixture of monoclinic WO_3_ and W_18_O_49_ with O/W = 2.98.

Surface roughness is higher for polycrystalline coatings (r > 1.5 nm) than for amorphous ones (r < 0.3 nm), because of the formation of large grains that clump together to form cauliflower-like clusters when the energy of the deposited particles increases. Otherwise, the bandgap energy is higher for amorphous films (E_g_ = 3.71 ± 0.1 eV) than for polycrystalline ones (E_g_ = 3.46 ± 0.1 eV), which is attributed to gap widening by quantum confinement in disordered samples.

All layers prepared at high oxygen pressures have low conductivity (σ~10^−4^ S/cm) and high transparency in the visible and near-infrared regions. Some coloration appears when the oxygen content decreases, due to sub-bandgap absorption by oxygen vacancy defects, which also increase the electrical conductivity. The samples with the lowest oxygen content (O/W ≤ 2.6) have a metallic behavior, with high conductivity (σ~10^1^ S/cm) and high Urbach energy (E_U_ > 1.5 eV), resulting from a large number of localized states within the bandgap region. For the other films, the Urbach energy is in a lower range E_U_ = 0.18–0.29 eV, being more dependent on the oxygen vacancy states than on the crystalline order.

## Figures and Tables

**Table 1:** Summary of deposition parameters and main characteristics of sputtered WO_3−x_ thin films.

Deposition Parameters		Atomic Composition	Structural Properties	Optical Properties		Electrical Properties
P (W/cm^2^)	O_pp_ (%)	O/W (at%)	XRD Data	E_g_ (eV)	E_U_ (eV)	σ (S/cm)
1	5	2.60	amorphous	metallic	1.79	1.45 × 10^1^
1	10	2.80	amorphous	3.72	0.29	7.85 × 10^−4^
1	15	2.98	amorphous	3.71	0.19	1.10 × 10^−4^
2	10	2.58	amorphous	metallic	1.57	1.05 × 10^1^
2	20	2.82	Orthorhombic WO_3_ & W_3_O_8_	3.47	0.21	9.10 × 10^−4^
2	30	2.98	Monoclinic WO_3_ &W_18_O_49_	3.45	0.18	1.20 × 10^−4^

## Data Availability

The data are available in this article.
